# Differing Tales of Two Patients after Receiving a Kidney Transplant from a Donor with Disseminated Intravascular Coagulation

**DOI:** 10.1155/2014/754256

**Published:** 2014-06-30

**Authors:** Pradeep V. Kadambi, Ann K. Gamilla-Crudo, Mohammad Almiani, Michelle A. Josephson, W. James Chon

**Affiliations:** ^1^University of Texas Medical Branch, 301 University Boulevard, Galveston, TX 77555, USA; ^2^University of Chicago, 5841 S Maryland Avenue, MC 5100, Chicago, IL 60637, USA

## Abstract

In order to decrease the time on the deceased donor kidney wait list and to have more organs available, criteria for acceptable organs for transplant could be made less stringent. There are reports of successful recipient outcomes using kidney donors presenting with disseminated intravascular coagulation (DIC). We report a unique circumstance where two patients received kidneys from the same deceased donor who had DIC; one patient developed thrombotic microangiopathy (TMA) while the other did not. This difference in outcome may indicate that both donor and recipient factors contribute to the development of posttransplant TMA.

## 1. Introduction

Kidney transplantation is the treatment of choice for end stage kidney disease (ESKD). Unfortunately, the wait time for a deceased donor kidney continues to increase every year as the number of potential recipients on the waiting list has far surpassed the number of kidney donors available for transplantation [1]. Over the past decade, initiatives such as the addition of Expanded Criteria Donors [2] and Donation after Cardiac Death [3] have had minimal impact on the number of available organs. Other strategies to increase organ availability are being undertaken. For example, a number of organs which were once thought unusable have been transplanted. We report our experience where at the time of recovery the donor had DIC, and the kidneys had TMA. Despite the microscopic findings, both kidneys were transplanted into different recipients with very different short-term clinical outcomes.

## 2. Case Report

The donor was a 24-year-old African American male with no significant medical history who sustained a gunshot wound to the head and subsequently developed DIC and multiorgan failure. The terminal serum creatinine was 6.0 mg/dL. Preimplantation biopsy of the donor's right kidney showed 27 glomeruli with no sclerosis; fibrin thrombi were present in 8 glomeruli; there was no interstitial fibrosis and tubular atrophy (IFTA), acute tubular necrosis (ATN), or infiltrates; mild (<25%) arterial sclerosis was identified but there was no arteriolar hyalinosis. Preimplantation biopsy of the left donor kidney revealed 34 glomeruli with 1 sclerosed glomerulus (2.9%); fibrin thrombi were present in 1 glomerulus; no IFTA, ATN, infiltrate, arterial, or arteriolar hyalinosis were observed.

The first recipient was a 55-year-old Caucasian male with ESKD secondary to hypertension who had been on hemodialysis for 6 years. There were 4 HLA mismatches with the donor, and the panel reactive antibody (PRA) titer at the time of transplant was 0%. He received the donor's right kidney. The cold ischemia time (CIT) was 18 hours and 10 minutes. The patient was induced with antithymocyte globulin and maintained on tacrolimus, mycophenolate sodium, and prednisone. He had an uncomplicated postoperative course with a serum creatinine of 3.1 mg/dL at time of discharge. A month later, the serum creatinine improved to 1.2 mg/dL.

The second recipient was a 39-year-old African American, nonobese female with body mass index of 28.7, with ESKD presumed to be due to hypertension, and had been on peritoneal dialysis for 9 years. She received the donor's left kidney and there were 4 HLA mismatches and the PRA was 0%. The CIT was 22 hours and 17 minutes. She was also induced with antithymocyte globulin. She had delayed graft function (DGF) requiring peritoneal dialysis for about 10 days after transplant.

As shown in Table 1, 48 hours after the transplant, she developed severe thrombocytopenia and anemia even before the calcineurin inhibitor (CNI) was started. A clinical diagnosis of thrombotic thrombocytopenic purpura-hemolytic uremic syndrome (TTP-HUS) was made based on the peripheral blood smear findings which revealed schistocytes and the laboratory values (high lactate dehydrogenase, low fibrinogen, and high fibrin split products). The ADAMTS13 activity was measured at 59%. On further questioning, the patient denied prior use of oral contraceptives or hormone replacement therapy.

She was treated with daily therapeutic plasma exchanges (total of 11 treatments) and four doses of rituximab (375 mg/m^2^ of body surface area or 700 mg per dose) on postoperative days 14, 21, 28, and 35. Her blood parameters improved with subsequent recovery of her kidney allograft as noted in Table 1.

## 3. Discussion

TMA refers to blood vessel wall thickening (mainly arterioles or capillaries) with swelling or detachment of the endothelial cell from the basement membrane, accumulation of fluffy material in the subendothelial space, intraluminal platelet thrombosis, and partial or complete obstruction of the vessel lumina [4]. Depending on whether kidney or brain lesions prevail, two pathologically indistinguishable but clinically separate entities occur: the hemolytic uremic syndrome (HUS) and the thrombotic thrombocytopenic purpura (TTP), respectively [5]. After kidney transplantation, TMA may be either recurrent or de novo. Among patients with ESKD due to TMA who undergo transplantation, the risk of recurrence depends upon the underlying etiology. Less commonly, patients who undergo renal transplantation for other causes of ESKD may also develop TMA; this is termed de novo TMA. Antibody mediated rejection (AMR) is a well-recognized cause of TMA after transplantation. It would be appropriate to check donor specific antibodies (DSA) upon recognition of clinical findings suggestive of TTP-HUS as TMA may often be found in the renal allograft.

The recurrence rate of TMA after kidney transplantation is reported between 25 and 50 percent [6, 7]. Most cases of recurrence are probably due to HUS and as previously mentioned the risk depends on the underlying cause of TMA. For example, recurrence among patients who had infection related variant of TMA (typical HUS) seems low. On the other hand, higher rates of recurrence are associated with the noninfection related or atypical variant of HUS (aHUS), where a clear link has been established to defects in regulation of the alternate complement pathway [8]. Mutations have been identified in complement factor H (CFH), complement factor I (CFI), matrix cofactor protein, and thrombomodulin [9]. In the right setting, these mutations may trigger TMA. While checking for complement factor mutations is ideal, the long turnaround time (~ 3 months) makes it impractical for rapid therapeutic decisions. In the appropriate clinical setting, empiric treatment with plasmapheresis is initiated as soon as the possibility of TTP-HUS is raised. Moreover specific genetic susceptibility mutations are identified in only about 60% of affected individuals.

De novo TMA after kidney transplantation is far less common with a reported incidence of only 0.8% in the analysis of United States Renal Data Systems [10]. Typically, it develops in the early posttransplant period; however, it may also be recognized years after transplant [11]. One of the most important risk factors for developing de novo TMA is the use of CNI as maintenance immunosuppression [12, 13]. The disease triggering effects of CNI seem multifactorial and are related to vasoconstriction, endothelial toxicity, and prothrombotic and antifibrinolytic actions. Other risk factors include viral infections (BK virus, parvovirus B19, and cytomegalovirus), use of Expanded Criteria Donor kidneys, presence of antiphospholipid antibodies, antibody mediated rejection, and other medications (clopidogrel and valacyclovir) [14–18].

DIC is characterized by activation of the coagulation pathway which results in the intravascular formation of fibrin [19]. This causes thrombotic occlusion of small and midsize vessels which can lead to multiple organ failure, including the kidneys [19]. This condition has been well described in patients with traumatic brain injury [20] who serve as a large source of deceased donors. Whether or not to accept kidneys for transplantation from donors with DIC, even from those who had normal kidney function prior to the DIC, is controversial. However, over the past few years, there is growing evidence of successful use of kidneys from such donors [21–23].

Supporting this approach were findings from a retrospective cohort of 162 kidney transplants in which donor DIC was not associated with suboptimal graft function in the short term [24].

The role of ADAMTS13 deficiency for identifying patients who have a clinical diagnosis of TTP has not been well defined. In one study, the presenting features and clinical outcomes of 16 patients with idiopathic TTP-HUS who had severe ADAMTS13 deficiency were variable and not distinctively different from the 32 patients with idiopathic TTP-HUS who did not have ADAMTS13 deficiency [25]. Regardless of ADAMTS13 activity, many patients responded to treatment with plasma exchange [25]. Patients described as having HUS were not [26] or were rarely [27] severely deficient in ADAMTS13 activity. Our patient had ADAMTS13 activity of 59%. Since there are no explicit criteria to distinguish patients with TTP from patients with HUS, the initial role of ADAMTS13 activity measurement remains unknown.

Our report indicates that both donor and recipient factors are important in posttransplant TMA as the first recipient did not manifest the disease while the second recipient did. Since the underlying cause of ESKD was reported as hypertension in the second recipient, donor DIC perhaps played an important role in causing TMA.

Organ transplantation by itself can cause microvascular injury in numerous ways and thus may trigger TMA [28]. Endothelial lesions in the graft, caused by prolonged warm ischemia, might increase antigenic presentation giving rise to acute rejection and TMA [14]. In a study of 24 patients with de novo posttransplant TMA, 7 (29%) carried mutations in CFH, CFI, or combined CFH/CFI indicating that undiagnosed complement abnormalities may represent important risk factors [29]. However, we did not check the second patient for complement mutations.

In general, the prognosis of de novo TMA is better than that of recurrent TMA. However, it may depend on the severity of clinical features and histological lesions. Patients with isolated glomerular TMA usually have a good outcome while patients with systemic signs and symptoms of TMA are more likely to need renal replacement therapy with associated loss of allograft function [11].

While there are no guidelines for treatment of de novo TMA after kidney transplantation, withdrawal of the offending agent is mandatory. Switching from a CNI based immunosuppressive regimen to a non-CNI based regimen can be helpful in some cases [30, 31]. Main therapeutic options include plasma exchange, intravenous immunoglobulin (IVIg), and rituximab [32–34].

Our second patient developed posttransplant TTP-HUS even before the initiation of CNI. The syndrome was recognized early and with aggressive treatment her clinical course improved. She was maintained on mycophenolate mofetil and prednisone and was started on tacrolimus after completion of plasma exchange with close monitoring of her clinical course. A biopsy was not performed after the intervention as the patient's kidney function remained stable. She did not have any subsequent episodes of TTP/HUS and her serum creatinine at 4 years after transplant was stable at 1.3 mg/dL.

More recently, there is evidence that eculizumab (anti-C5a) may be effective in treating and preventing recurrence of aHUS after kidney transplantation [35, 36].

## 4. Conclusion

TMA in either the kidney recipient or the donor is generally associated with a poor prognosis. While donor kidneys with TMA should not be automatically discarded, reasonable caution should be exercised as to whom these kidneys should be transplanted into. For example, they should not be used in recipients who have had previous episodes of TMA needing therapy or those who currently have thrombocytopenia for unknown reasons. Needless to say, informed consent is critical. Recipients should be informed about the potential risk of developing TMA after transplant with associated loss of the allograft. Patients should be monitored closely after transplant and therapy instituted as soon as possible if there are signs or symptoms of TMA, either clinically or on the allograft biopsy. While conventional therapies of plasma exchange, IVIg and rituximab, are frequently used, the anti-C5a antibody eculizumab offers promise.

## Figures and Tables

**Table 1:** Pertinent laboratory values of the second patient describing the postoperative clinical course.

Labs	Before surgery	48 hours after transplant	4 weeks later/at discharge	2 years after transplant
WBC (×1000/L)	10	8.6	5.2	6
Hemoglobin (gm/dL)	8.6	7.1	11	11.2
Platelets (×1000/L)	289	35	239	188
Prothrombin time (sec)	14	16		
Partial thromboplastin time (sec)	30	32		
Lactate dehydrogenase (mg/mL)		622		
Haptoglobin (mg/dL)		<20		
Coombs' test		Negative		
ADAMTS13 activity		59%		
HIT antibody PF-4 assay∗		Negative		
Creatinine (mg/dL)	14	12.5	1.4	1.3

*HIT: heparin induced thrombocytopenia; PF-4: platelet factor 4.
